# The Effect of Physical Activity in Parkinson’s Disease: A Mini-Review

**DOI:** 10.7759/cureus.2995

**Published:** 2018-07-18

**Authors:** Muniba Fayyaz, Syeda S Jaffery, Fatima Anwer, Ahsan Zil-E-Ali, Ibrar Anjum

**Affiliations:** 1 Internal Medicine, Fatima Memorial Hospital, Lahore, PAK; 2 Research, CiBNP, Fairfield, USA; 3 Medicine, Mayo Hospital King Edward Medical College, Lahore, PAK; 4 General Surgery, Fatima Memorial Hospital, Lahore, PAK; 5 Internal Medicine, The University of Texas MD Anderson Cancer Center, Houston, USA

**Keywords:** parkinson's disease, physical activity, cognition, tremors, pain, motor

## Abstract

This article will discuss the effects of physical activity in patients with Parkinson’s disease. Presently, the motor and non-motor symptoms are difficult to treat with the current treatment of Parkinson's; therefore, as an adjuvant to the current treatment physical activity, has been recommended. Physical activity has been known to improve many symptoms in patients with Parkinson's disease. Some of these symptoms include the physical capacities, physical and cognitive functional capacities. Physical activity also slows the disease process, decreases the pain associated with Parkinson`s disease, prolongs the independent mobility (gait, balance, strength) and improves sleep, mood, memory hence improving the overall quality of life. Furthermore, physical activity has the potential to improve the non-motor symptoms (depression, apathy, fatigue, constipation) and the secondary complications of immobility (cardiovascular, osteoporosis) in Parkinson's disease.

## Introduction and background

Parkinson’s disease (PD) is a chronic progressive neurodegenerative disease first described by Dr. James Parkinson in 1817 as a “shaking palsy” [1]. Its prevalence is estimated at 2,802 per 100,000 persons in North America, Europe, and Australia [2]. It is accompanied by both motor and non-motor symptoms due to the loss of striatal dopaminergic and non-dopaminergic neurons [1]. The symptoms of Parkinson’s disease include tremors, bradykinesia, rigidity, postural instability, altered gait pattern, freezing of gait, and motor coordination deficits. Other symptoms such as cognitive impairment, dementia, insomnia, depression, anxiety, apathy, bladder dysfunction, pain, and fatigue can also occur [2]. Parkinson’s disease is a highly variable disease and the symptoms can greatly affect a person’s quality of life, limit functional capacities, daily activities and social interactions [1]. Since there are no definite tests for Parkinson’s disease, a physician can diagnose it by using a comprehensive history and physical examination [1]. The current treatment of Parkinson’s consists of medical therapies (levodopa, catechol-O-methyl transferase (COMP) inhibitors, dopaminergic/non-dopaminergic drugs), non-pharmacological therapy and neurosurgical (deep brain stimulation) [3]. Research has suggested that physical activity (PA) can also be added to the therapy to limit disability and improve the quality of life. Many articles have discussed the effects of physical activity on the symptoms of Parkinson’s disease in the past. In this article, we will combine the results of different articles in a summarized form and discuss how physical activity can affect the different symptoms of Parkinson’s disease.

## Review

Parkinson's disease (PD), the most common neurodegenerative disease of the elderly, is characterized by the progressive loss of muscle control. Some studies show the effects of physical activity occurring on the different symptoms of Parkinson’s disease. First, Jankovic et al discussed that physical activity had the most effective improvement on the physical capacities and physical and cognitive functional capacities in patients with PD. On the other hand, physical activity seemed less efficient at improving clinical symptoms of PD and psychosocial aspects of life, with only 50% or less of results reporting positive effects. The impact of PA on cognitive functions and depression also appeared weaker and more research was recommended for the effects of PA on cognitive functions, depression and specific symptoms of PD [3]. In contrast, Murray et al. discussed the positive effects of exercise on cognition and obtained evidence from animal studies supporting the role of exercise to improve cognition in humans through the promotion of neuronal proliferation, neuroprotection, and neurogenesis. They combined various studies showing that different kinds of exercise, including aerobic, resistance, and dance can improve cognitive function; however, the type, amount, mechanisms, and duration of exercise remained unclear [4]. Also, Ahlskog JE discussed that the result of physical activity has not only been associated with better cognitive scores, but midlife exercise significantly reduces the later risk of both dementia and mild cognitive impairment. He also demonstrates that exercise during midlife decreases the risk of developing PD and emphasized that ongoing vigorous exercise/physical activity helped in decreasing the medical refractory motor problems of PD. In his experiment, the animal models exercise increased BDNF (brain-derived neurotrophic factor) that provided protection from the dopaminergic neurotoxins by mediating brain neurotrophic factors and neuroplasticity. Exercise consistently improved cognition in animals and this was also linked to enhanced neuroplasticity and increased neurotrophic factor expression. In these animal models, immobilization had the opposite effect. He concluded that brain-derived neurotrophic factor (BDNF) may mediate at least some of this exercise benefit. In humans, exercise also increased serum BDNF which is known to cross the blood-brain barrier [5]. Physical activity is also known to prolong independent mobility and improves sleep, mood, memory, and quality of life, all further enhanced through socialization and multidisciplinary team support [6]. Optimally prescribed exercise program following diagnosis may alter neurophysiological processes, possibly slowing symptoms [4].

In addition, Kolk et al. discuss the balance and gait deficits in Parkinson's disease are especially difficult to treat by the current pharmacological or surgical treatment. There study shows that exercise has the potential to help both motor (gait, balance, strength) and nonmotor (depression, apathy, fatigue, constipation) aspects of Parkinson's disease as well as secondary complications of immobility (cardiovascular, osteoporosis) [7]. Moreover, a study conducted by Park et al. concluded that long-term, group exercise programs are possible in the Parkinson's disease population, with excellent adherence and minimal dropout. Even though their outcome measures did not provide strong evidence that exercise has a neuroprotective effect on motor function, the group with an earlier participation in exercise showed a significant effect on symptoms of depression [8]. Moreover, Cholewa et al. performed a study in 2013, in which 70 patients with PD took part in 60-minute rehabilitation exercises twice a week, which aimed at increasing movement ranges, balance improvement, movement agility and walking. It concluded the reduction of the severity of motor symptoms in patients with PD when enhancing their daily activities [9]. Also, Moroz et al. discusses the functional deterioration in patients with PD and elaborated that the role of caregivers is very critical, thus training caregivers is of paramount importance to help maintain a safe environment and limit caregiver anxiety and depression [10]. A case report by Kluding et al., illustrated two 66-year-old males, both with Parkinson's disease, participated in three months of various physical activities. After the three-month program, improvements were noted for both individuals in functional reach, Timed Up and Go, and Berg Balance scores. Following the program, both participants continued to exercise regularly for at least 8 months [11]. Furthermore, a randomized clinical trial tested the differences in three types of exercises for Parkinson patients. In their study, they tested treadmill, resistance, and stretching exercise on gait speed, strength and, fitness. They concluded that lower-intensity treadmill exercise resulted in the maximum improvement in gait speed. Also, the cardiovascular fitness was improved in both the higher- and lower-intensity treadmill exercises. Out of the three exercises, only the stretching and resistance exercises improved the muscle strength. Overall, combining the treadmill and resistance exercises may produce a greater result [12]. 

The effect of physical activity on pain in patients with Parkinson's disease

The effects of physical activity on pain has also been emphasized in the past. Allen et al. discuss that pain is a common symptom experienced by a Parkinson patient and occurs in 85% of patients. It is associated with a lack of sleep, depression and a decrease in the overall quality of life. Even though pain is a very distressing symptom, it usually receives less attention. This could be due to many reasons such as the complex nature of pain, the decrease in understanding of its neurophysiology and the lack of clinical trials in the management of pain in patients with Parkinson's disease. The pain in Parkinson disease can be due to several factors which include the disease process, Parkinson disease impairments as well as co-existing musculoskeletal and/or neuropathic pain conditions. The pain can be controlled by the conventional analgesics and with the use of dopaminergic agents. This study emphasized that physical activity can affect pain greatly by contributing to neuroplasticity (anatomical, physiological and functional re-organisation of the brain in response to changes in environment/behaviour) and neuro-restoration (regeneration of damaged nervous tissue) by increasing brain neurotrophic factors, synaptic strength and angiogenesis, as well as stimulating neurogenesis and improving metabolism and the immune response. These changes may be beneficial in improving the central processing of pain. This study also highlights that exercise can activate the dopaminergic neurons that have an anti-nociceptive role, suppressing nociceptive signaling in dorsal root ganglion and modulating pain in rostro-ventral medulla. Lastly, exercise can help reduce the pain in Parkinson's by activating the non-dopaminergic pain inhibitor pathways (all of which are degenerated in Parkinson's) which include noradrenergic, serotonergic, cholinergic, and peptidergic systems that are essential systems for pain processing and descending pain inhibition [13].

## Conclusions

In conclusion, Parkinson disease is a disabling neurodegenerative disease that can greatly affect a person’s overall quality of life. The motor and nonmotor symptoms remain difficult to manage with current clinical therapies, but exercise has been identified as a possible adjuvant treatment and can be considered to help improve a person’s limitations and improve the daily activities. It is important that physicians keep this treatment in mind while treating patients with Parkinson’s disease.
